# Light-Irradiation Wavelength and Intensity Changes Influence Aflatoxin Synthesis in Fungi

**DOI:** 10.3390/toxins10010031

**Published:** 2018-01-05

**Authors:** Tadahiro Suzuki

**Affiliations:** Division of Food Biotechnology, Food Research Institute, NARO, 2-1-12 Kannon-dai, Tsukuba, Ibaraki 305-8642, Japan; suzut@affrc.go.jp; Tel.: +81-29-838-8063

**Keywords:** light wavelength, intensity, aflatoxin, mycotoxin, This study represented that differences of light irradiation setting through changes of wavelength and intensity influence aflatoxin synthesis. Blue-green low intensity irradiation was the most effective condition for the aflatoxins synthesis.

## Abstract

Fungi respond to light irradiation by forming conidia and occasionally synthesizing mycotoxins. Several light wavelengths, such as blue and red, affect the latter. However, the relationship between light irradiation and mycotoxin synthesis varies depending on the fungal species or strain. This study focused on aflatoxin (AF), which is a mycotoxin, and the types of light irradiation that increase AF synthesis. Light-irradiation tests using the visible region indicated that blue wavelengths in the lower 500 nm region promoted AF synthesis. In contrast, red wavelengths of 660 nm resulted in limited significant changes compared with dark conditions. Irradiation tests with different intensity levels indicated that a low light intensity increased AF synthesis. For one fungal strain, light irradiation decreased the AF synthesis under all wavelength conditions. However, the decrease was mitigated by 525 nm low intensity irradiation. Thus, blue-green low intensity irradiation may increase AF synthesis in fungi.

## 1. Introduction

Fungi have various effects on humans, animals, and plants. Some fungi are used in fermentation, brewing, and vinification. Additionally, a number of antibiotic agents have been discovered from fungi. However, fungi also infect humans, animals and plants, causing various diseases and resulting in illness and economic losses [1,2,3]. It is difficult to regulate individual fungi because they are distributed across a range of conditions, although fungi used for fermentation are well controlled in limited artificial environments. Thus, identifying conditions that decrease contamination will increase crop yields and reduce the harm to animals and humans. Additionally, developing new techniques to control fungal species may lead to the development of faster and more effective food-processing methods.

The sensitivity of *Chlamydomonas reinhardtii* to some trichothecene mycotoxins produced by *Fusarium* species is influenced by changes in light intensity [4]. This algal species has general plant mechanisms, such a light-dependency, that are also observed between fungi and host plants. Fungi respond to light irradiation by changing physiologically. For instance, blue-light irradiation influences conidiation in *Neurospora crassa*, *Bipolaris oryzae*, and *Trichoderma atroviride* [5,6,7,8,9], and also influences the circadian rhythms of *N. crassa* [10]. *B. oryzae* has blue-light receptors named *BLR1* and *BLR2*, and *N. crassa* has homologous receptors named *WC-1* and *WC-2*. Such receptors are also conserved in *Aspergillus nidulans*, *Aspergillus fumigatus* and *Aspergillus oryzae* [11,12,13,14]. In addition, species with red-light receptors have been reported [11,13]. Therefore, it is highly possible that the blue-light response system is conserved among various fungal strains. This system activates conidial formation, and its receptors act as transcription factors [14]. Blue light also inhibits mycelial growth. *A. oryzae* forms conidia in response to white-light irradiation and is inhibited by red-light irradiation [14]. However, Schmidt-Heydt et al. [15] reported that blue-light irradiation inhibits the conidial formation of some *Aspergillus* strains. Additionally, blue-light irradiation inhibits the mycelial growth of *Aspergillus carbonarius* and *Aspergillus westerdijkiae* [16]. Thus, light irradiation influences fungal metabolism but the effects are complex.

Light irradiation affects not only primary metabolism but also secondary metabolism. Indeed, *A. carbonarius* and *A. westerdijkiae*, which produce the mycotoxin ochratoxin A (OTA), are affected by light irradiation, and OTA synthesis is changed [16]. OTA synthesis is increased by red-light irradiation in an *A. carbonarius* strain, but is decreased in an *A. westerdijkiae* strain. However, the OTA synthesis of *A. westerdijkiae* is also decreased by continuous daylight irradiation, which has a yellow to orange peak wavelength. Thus, interpreting the results is not simple. *Aspergillus parasiticus* BFE96p decreases the synthesis of the mycotoxin aflatoxin (AF) under both blue- and red-light conditions [15]. Additionally, *Fusarium graminearum* BFE1006 and *Penicillium expansum* BFE189 increase deoxynivalenol and citrinin, which are mycotoxins, during blue-light irradiation, respectively. Thus, the relationship between light irradiation and physiological mechanisms is complicated because the testing conditions and fungal strains used are varied. Therefore, unified testing conditions are required to interpret the influence of light irradiation.

Here, specific wavelength settings and four fungal strains were used, and the effects on one metabolite were assessed. AF was selected as the target metabolite because AF contamination is a major risk [17,18,19] and information on its regulatory mechanism is urgently required.

## 2. Results

### 2.1. Visible Changes of Plate Cultures Caused by Conidiation and Fluorescence

To observe the influence of light irradiation at wavelengths at both ends of the visible region, 401 nm and 470 nm blue light, and 660 nm and 720 nm red light, were used to independently irradiate fungal strains on culture plates. White light, which has blue and yellow peaks (Figure S1), was also used. To establish a uniform environment for different light radiation levels, the photon flux density (PFD) was set to 4 μmol·m^−2^·s^−1^ under all light conditions.

**Figure 1:**
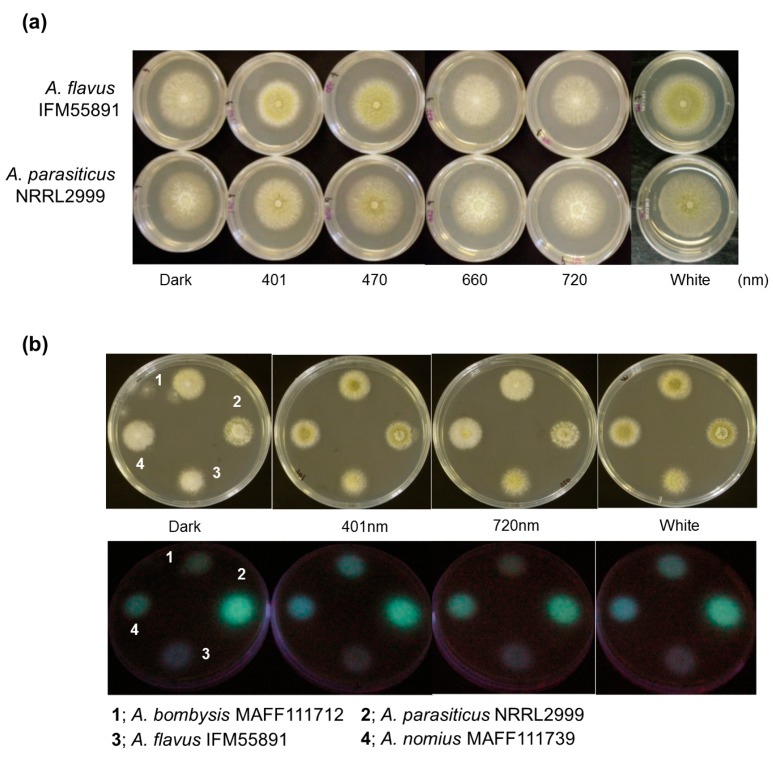
Fungal colonies cultured under different light conditions. (**a**) Differences in conidial formation. Each strain was incubated on potato dextrose agar (PDA) plates under continuous light with the PFD set at 4 μmol·m^−2^·s^−1^, except in the dark controls. Yellow-green colonies were observed under 401 nm, 470 nm and white light, indicating conidial formation; and (**b**) differences in aflatoxin-derived fluorescence intensities among different light conditions. Four strains were placed on PDA plates containing 0.3 g·L^−1^ carbon and 3 g·L^−1^ α-cyclodextrin and incubated under different light conditions, and then the undersides of the culture plates were irradiated using 365 nm ultraviolet light.

First, to confirm the effects of light on conidial formation, spores of two fungal strains, *Aspergillus flavus* IFM55891 and *A. parasiticus* NRRL2999, were spotted onto potato dextrose agar (PDA) plates and incubated for 2–3 days. To compare colony formation, samples were also grown under dark conditions as fungal controls. Dark conditions resulted in white colonies with limited conidial formation (Figure 1a). Under red-light conditions, both strains produced white colonies similar to the controls. However, when both fungal strains were grown under blue- and white-light conditions, yellow-greenish conidia formed.

Since blue- and red-light conditions resulted in different fungal colony phenotypes, their effects on AF synthesis were investigated. Irradiation conditions were set using wavelengths at both ends of the visible region (401 nm blue light and 720 nm red light), and the light intensity was the same as in the conidial formation test. Additionally, since light irradiation caused changes in fungal phenotypes regardless of the strain, *Aspergillus bombysis* MAFF111712 and *Aspergillus nomius* MAFF111739 were added to this test. The observational test of AF-derived fluorescence was conducted using PDA containing α-cyclodextrin (α-CD) and activated carbon to increase the visibility of the fluorescence [20,21,22]. AF-derived fluorescence was observed from the undersides of the culture plates. The colonies of *A. bombysis* MAFF111712 and *A. nomius* MAFF111739 had increased fluorescence levels under 401 nm irradiation compared with the control dark condition (Figure 1b). The 720 nm irradiation condition resulted in the same level of fluorescence as the dark condition. However, *A. flavus* IFM55891 did not show any intensity-derived changes, while *A. parasiticus* NRRL2999 had a decreased fluorescence level.

**Figure 2:**
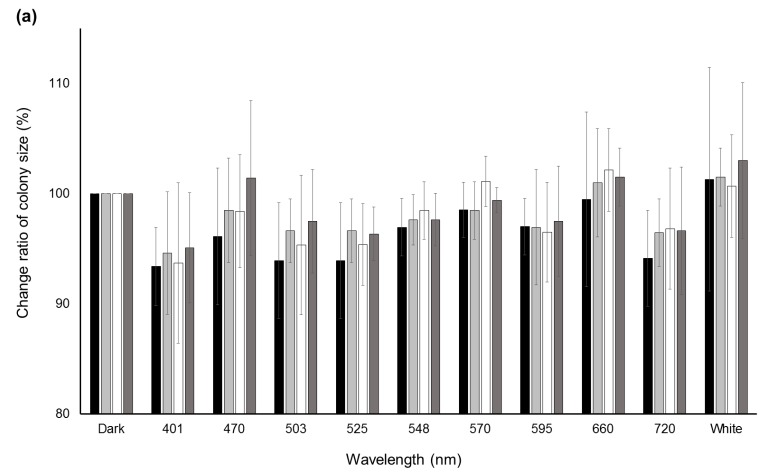

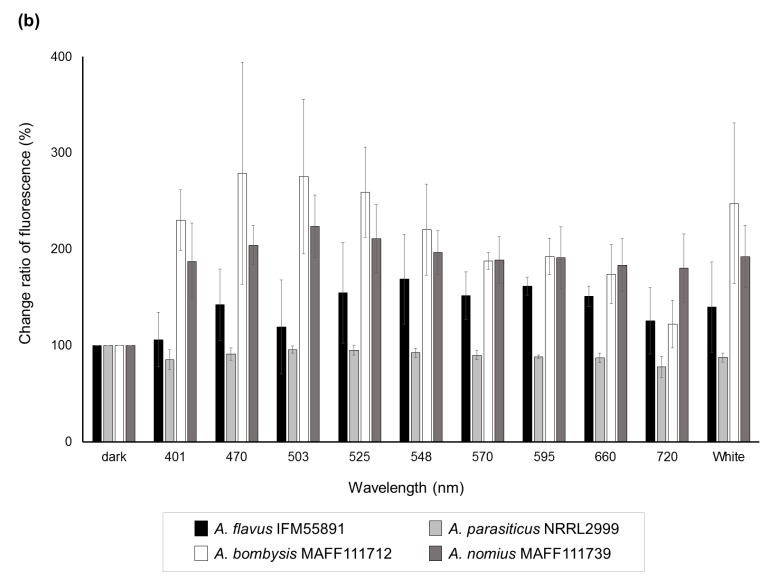
Change ratios of colony size and AF-derived fluorescence among different light conditions. (**a**) Changes in colony size after a two-day incubation under different light conditions. Colony sizes after incubation under dark conditions were standardized as the control; (**b**) AF-derived fluorescence obtained by UV irradiation of the undersides of the culture plates. Fluorescence intensities obtained from dark conditions were standardized as the control. Significant increases (*p* < 0.05); 548–660 nm for *A. flavus* IFM55891, 401 and 503–660 nm for *A. bombysis* MAFF111712, 470–660 nm and white for *A. nomius* MAFF111739. Significant decreases (*p* < 0.05); 570–660 nm and white for *A. parasiticus* NRRL2999. Bars indicate standard deviations; *n* = 3.

Significant decreases (*p* < 0.05) in the change ratio of colony size were observed at 401 nm for *A. flavus* IFM55891 and 525 nm for *A. bombysis* MAFF111712 and *A. nomius* MAFF111739 compared with their respective controls under dark conditions (Figure 2a). However, continuous or wavelength-dependent changes were not observed. Measurements of AF-derived fluorescence under a wide range of wavelength conditions indicated that light irradiation increased the fluorescence intensity regardless of wavelength, except in *A. parasiticus* NRRL2999 (Figure 2b). In particular, during irradiation in the blue to green region (470–548 nm), the AF-derived fluorescence levels of *A. flavus* IFM55891, *A. bombysis* MAFF111712 and *A. nomius* MAFF111739 were at their highest. The intensity levels under white-light conditions were the next highest, and even irradiation using the red-light region resulted in higher fluorescence intensities than dark conditions.

### 2.2. Measurement of Aflatoxins in Liquid Cultures

The AF-derived fluorescence intensity levels of *A. parasiticus* NRRL2999 did not show significant differences in the plate test, although other fungal strains showed increased intensity levels as a result of light irradiation. Moreover, the peak radiation regions that caused the highest fluorescence intensity levels differed among the three fungal strains. Thus, the most effective wavelength for AF synthesis was not clear. To solve this problem, the AF volume was measured using a micropipette tip-based liquid culture method [23] (Figure S2). To correspond to the plate culture test, the method used liquid PD medium containing activated carbon. While adding α-CD to the culture plate does not increase AF synthesis in fungi, AF inclusion products increase the fluorescence intensity through an optical effect [24,25]; thus, α-CD was not added to the liquid culture to avoid contamination with AF inclusion products. To make the liquid culture conditions close to the plate culture conditions, the incubation time was set to three days, and the same four fungal strains were used. The light conditions were also the same as for the culture plate test.

Slight decreases at 401 nm were observed for the fungal masses formed in each pipette tip, although wavelength-dependent changes were not observed (Figure S3). After collecting the culture medium, four types of AF (AFB_1_, AFB_2_, AFG_1_, and AFG_2_) were measured (Table S1). Under the single-wavelength irradiation conditions between 470 nm and 548 nm, all of the fungal strains displayed maximum AF volumes. Compared with the dark conditions, *A. nomius* MAFF111739 had an increased AF-synthesis level at 401 nm, while the other strains showed decreased levels.

Furthermore, AF synthesis decreased or changed insignificantly from 570 to 660 nm, except in *A. nomius* MAFF111739. The amount of AF synthesis under white-light conditions was similar to that from 470 to 548 nm. When compared with dark conditions, the AF synthesis in *A. parasiticus* NRRL2999 decreased, except under bluish- and white-light conditions.

**Figure 3:**
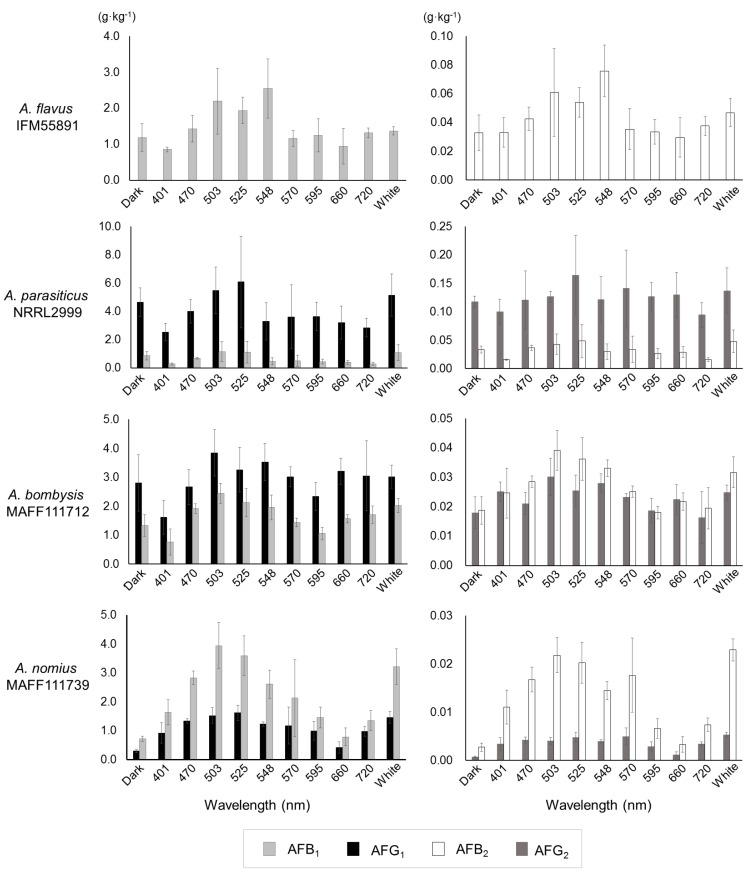
Aflatoxin concentrations per unit fungal mass (g·kg^−1^) obtained by integrating the fungal dry mass (Figure S3) and aflatoxin amounts (Figure S4). Bars indicate standard deviation; *n* = 3–6.

The AF concentrations calculated based on the AF amounts per unit fungal dry mass also indicated that bluish irradiation resulted in maximum values, and the results corresponded to the AF synthesis trend (Figure 3). Statistical analyses were conducted to confirm how AF synthesis changed at each irradiation level (Table 1). The dark-condition samples were set as controls, and the significant differences at each wavelength were identified by conducting an unpaired or Welch’s *t*-test. Irradiation in the lower 500 nm region, focused on 525 nm, caused a significant increase in AF synthesis. The number of significant changes decreased in the higher end of the 500 nm region, the fewest significant changes were observed at 660 nm. The number of significant differences under white-light conditions followed that of the lower 500 nm region.

### 2.3. Exposure to Different Irradiation Intensity Levels Results in Varied Aflatoxin Synthesis

In this study, statistical analyses indicated that 525 nm irradiation induced the maximum AF synthesis level and that 660 nm irradiation caused few significant changes. Past studies investigating the relationship between light irradiation wavelengths and mycotoxin production have not produced unified results [15,16]. One explanation for these differences may be the use of different fungal strains. Additionally, differences in conditions other than wavelength, such as the irradiation intensity, might have influenced the efficiency of AF synthesis. Initially, a 4-μmol·m^−2^·s^−1^ intensity setting was used in this study, which was low compared with that used in past studies. Thus, another test was conducted using an intensity of 40 μmol·m^−2^·s^−1^ at 525 nm and 660 nm, and the effects on AF synthesis were compared with those at 4 μmol·m^−2^·s^−1^.

**Figure 4:**
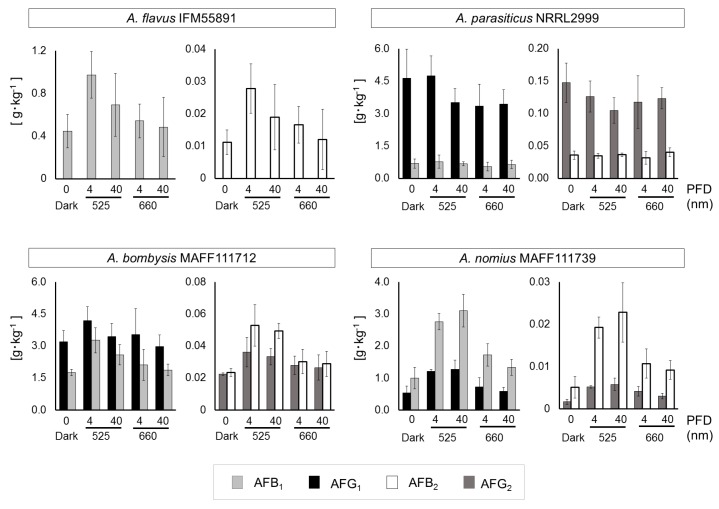
Aflatoxin concentrations per unit fungal mass (g·kg^−1^) that were obtained by integrating the fungal dry mass (Figure S5) and aflatoxin amounts (Figure S6). Light intensities of 4 and 40 μmol·m^−2^·s^−1^ at 525 and 660 nm were used, except under dark conditions. Each sample was newly prepared for the comparison study. Significant increases (*p* < 0.05); 4 μmol·m^−2^·s^−1^ at 525 nm conditions: AFB_1_ and AFB_2_ for *A. flavus* IFM55891, AFB_1_, AFB_2_, and AFG_2_ for *A. bombysis* MAFF111712, all AFs for *A. nomius* MAFF111739; 40 μmol·m^−2^·s^−1^ at 525 nm conditions: AFB_1_, AFB_2_, and AFG2 for *A. bombysis* MAFF111712, all AFs for *A. nomius* MAFF111739; 4 μmol·m^−2^·s^−1^ at 660 nm conditions: AFB_1_, AFB_2_, and AFG_2_ for *A. nomius* MAFF111739; 40 μmol·m^−2^·s^−1^ at 660 nm conditions: AFG_2_ for *A. nomius* MAFF111739. Bars indicate standard deviation; *n* = 4.

Fungal growth levels and mycelial masses remained unchanged, except the masses of *A. flavus* IFM55891 and *A. nomius* MAFF111739 under dark conditions (Figure S4). For *A. flavus* IFM55891, AF synthesis under light-irradiation conditions was greater than under dark conditions, regardless of the irradiation intensity or wavelength (Table S2). According to calculations of the AF amounts per unit fungal dry mass, 4 μmol·m^−2^·s^−1^ at 525 nm conditions resulted in the highest level of AF synthesis (Figure 4). The results for *A. bombysis* MAFF111712 were similar. *A. nomius* MAFF111739 had the highest AF synthesis level under 40 μmol·m^−2^·s^−1^ at 525 nm conditions, although other irradiation-condition results corresponded to those of *A. flavus* IFM55891 and *A. bombysis* MAFF111712. Only *A. parasiticus* NRRL2999 differed from the other strains, with the highest AF synthesis occurring under dark conditions. Overall, the 4 μmol·m^−2^·s^−1^ irradiation intensity increased AF synthesis more than 40 μmol·m^−2^·s^−1^ irradiation, although there were exceptions.

## 3. Discussion

### 3.1. Blue-Light Irradiation Facilitates Conidial Formation under Low-Intensity Conditions

The relationship between light irradiation and fungi has been investigated, and the most obvious phenotypical change is in conidial formation [14,15]. The light-response mechanism related to conidial formation has become clear [12,26], and may be distributed throughout fungi. However, different responses to the same color of light radiation have been reported in different fungal strains [15,16]. Therefore, knowing the phenotypic characteristics of test strains is a preliminary requirement for light-irradiation studies. Here, the light-irradiation test indicated that blue-light conditions of 401 nm and 470 nm irradiation stimulated conidial formation, and white-light conditions also resulted in conidial formation (Figure 1a). This implies that conidial formation is not prevented under irradiation conditions by specific light wavelengths if there is a component of the blue-light region present. These trends were also seen in *A. flavus* IFM55891 and *A. parasiticus* NRRL2999, confirming that this was not a specific condition. Previous studies have presented partially corresponding and differing data compared with those of our study [14,15]. However, these studies used different strains or irradiation conditions, making direct comparisons difficult.

### 3.2. Fungal Plate Cultures Undergoing Light Irradiation Provide an Improved Detection Technique for Aflatoxin Synthesis

Since conidial formation represents a large physiological change, blue-light irradiation should affect AF synthesis. Clarifying the mechanism will be useful in regulating AF synthesis. Therefore, the relationship between light irradiation and AF synthesis was investigated. Using fungal plate cultures with light irradiation increased the observational efficiency for AF-derived fluorescence from *A. bombysis* MAFF111712 and *A. nomius* MAFF111739 under 401 nm irradiation conditions compared with dark conditions (Figure 1b). The fluorescence intensity levels of *A. bombysis* MAFF111712 and *A. nomius* MAFF111739 induced after a few days of incubation were not originally at high levels. Thus, it was expected that the blue-light irradiation would effectively increase the AF-derived fluorescence observed from the plate cultures. However, increased fluorescence intensity after blue-light irradiation was not observed for *A. flavus* IFM55891 or *A. parasiticus* NRRL2999. Thus, it is difficult to determine whether the different irradiation conditions induced the changes in fluorescence intensity levels. For white-light irradiation, both conidial formation and increased fluorescence intensity were observed, similar to the blue-light irradiation conditions. These changes correlate, on the whole, with those of the blue-light components. However, for *A. nomius* MAFF111739, 720 nm red-light irradiation increased the AF fluorescence despite the low level of conidial formation. In contrast, for *A. flavus* IFM55891 and *A. parasiticus* NRRL2999, 401 nm blue-light irradiation increased conidial formation, even though AF-derived fluorescence intensity levels were not increased. The test of specific wavelengths also showed a similar trend (Figure 2b). These different phenotypic changes among fungal strains suggest that the regulation of AF synthesis and conidial formation are separately influenced by light irradiation, although there is a commonality in that the DNA methyltransferase protein contributes to both [26]. Three strains showed fluorescence intensity maximums under blue-green-light conditions (Figure 2b). However, the irradiation wavelengths that induced the highest values were slightly misaligned and, therefore, a specific wavelength that increases AF synthesis the most in all fungal strains could not be determined. This paradoxically suggests that it is not necessarily the specific environment, including the applied single-wavelength irradiation condition that results in increased AF synthesis. The addition of general blue- or white-light irradiation to the culture conditions may help us to identify AF-synthetic fungi if the fungal strain has a low AF-synthesis level or it is hard to recognize this characteristic.

### 3.3. Low-Intensity Bluish-Green-Light Irradiation Is More Effective in Increasing AF Synthesis than High-Intensity Irradiation or Other Wavelength Conditions, Although This Is Not Universal among Fungi

In this study, a liquid culture test was conducted to quantitate the levels of AF synthesis (Table S1, Figure 3). Slight changes that were hard to discriminate by direct observation using plate cultures were observed for *A. flavus* IFM55891 and *A. parasiticus* NRRL2999, and bluish-green-light irradiation composed of the lower half of the 500 nm region increased AF synthesis. At the same time, the AF synthesis of *A. parasiticus* NRRL2999 decreased, except under 500 nm irradiation. These changes in AF amounts suggested that exposure to light, except bluish-green-light irradiation, resulted in the suppression of AF synthesis. This trend was also reported in a previous test using *A. parasiticus* BFE96p under green-light conditions [15]. White light, which contains blue- and yellow-wavelength regions, showed the same synthesis trend as 500 nm irradiation. Thus, the liquid culture test supports that bluish-green- and white-light irradiation are useful for increasing or maintaining AF synthesis. In particular, 525 nm irradiation may be the most effective condition for AF synthesis (Table 1). However, blue-light irradiation is not always useful in increasing mycotoxin synthesis [15,16]. Thus, the results of this study do not entirely correspond to those of previous studies. However, different conditions were applied in each study, including light intensity, which we adjusted in this study. First, we used 4 μmol·m^−2^·s^−1^ as the low-intensity condition, which may be preferred by fungi, although other reports have used higher intensities. Then, we applied a high-intensity setting of 40 μmol·m^−2^·s^−1^, and the results were compared with those of the low-intensity condition. The low-intensity setting resulted in increased AF synthesis compared with the 40-μmol·m^−2^·s^−1^ high-intensity condition (Table S2, Figure 4). In particular, the AF synthesis of *A. parasiticus* NRRL2999 at 40 μmol·m^−2^·s^−1^ was similar to that under red-light conditions, and the synthesis at 4 μmol·m^−2^·s^−1^ was similar to that under the dark condition.

These trends were different from those of other strains. Thus, *A. parasiticus* NRRL2999 might have a different light-response system. *A. bombysis* MAFF111712 showed a relatively small change in AFG_1_. All of the AF fractions of *A. parasiticus* NRRL2999 and AFG_1_ of *A. bombysis* MAFF111712, which were not greatly increased by 525 nm irradiation, were synthesized in large amounts under the dark condition compared with the other strains. This suggested that 525 nm irradiation is useful for strains that produce small amounts of AF under dark conditions. In contrast, it may have an adverse effect on strains that synthesize large amounts of AF. The fungal light-response system is regulated through a mitogen-activated pathway, which also regulates some stress responses [12]. Considering this mechanism, high amounts of AF in the culture media might act as a stressor. The relationship between the AF-synthesis mechanism and light responses may vary, and the results of this study will help to interpret how light stimuli influences AF synthesis. This study used a three-day incubation period for both plate and liquid culture tests to unify the incubation conditions and, therefore, the influence of a longer incubation period is unknown. The results with the three-day incubation period are different from those of *A. carbonarius*, in which OTA was increased by red-light irradiation [16]. In contrast, this study implies that the blue-light stimulus is related to the AF-synthesis mechanism. The red-light stimulus resulted in only small changes in AF synthesis, suggesting that the relationship between blue-light irradiation and AF synthesis is more important. However, even under red-light conditions, an increase in AF synthesis was observed, except in *A. parasiticus* NRRL2999. Therefore, it is unlikely that the increase is regulated by the blue-light response mechanism alone. In fact, AF synthesis in *A. parasiticus* BFE96p and OTA synthesis in *A. westerdijkiae* are repressed by both blue- and red-light irradiation [15,16]. However, the irradiation conditions used in those studies were not consistent. Therefore, the trends of AF and OTA synthesis might have changed owing to other irradiation conditions. Like *A. parasiticus* BFE96p, the AF level produced by *A. parasiticus* NRRL2999 at 40 μmol·m^−2^·s^−1^ decreased, whereas *A. parasiticus* NRRL2999 maintained its AF level at 4 μmol·m^−2^·s^−1^. This suggests that the light intensity, like the wavelength, is important. Based on the four strains used here, the low-intensity condition is more suitable for AF synthesis.

## 4. Conclusions

The physiological behaviors of fungal strains in response to light vary depending on the light conditions or fungal strains used. Thus, a mechanistic link between mycotoxin synthesis and light irradiation cannot be presumed. However, the changes observed in our, and previous, studies suggest that light stimuli affect mycotoxin synthesis. Clarifying this situation will help develop a regulatory method to inhibit mycotoxin synthesis by influencing the physiological processes of fungi.

## 5. Materials and Methods

### 5.1. Fungal Species

Fungal strains were obtained as follows: *A. flavus* IFM55891 (NBRC 33021, ATC C22546) from Chiba University’s Medical Mycology Research Center (Chiba, Japan), *A. parasiticus* NRRL2999 from Prof. Kimiko Yabe at the Faculty of Environmental and Information Science, Fukui University of Technology (Fukui, Japan), *A. bombysis* MAFF111712 and *A. nomius* MAFF111739 from the Genebank Project, National Agriculture and Food Research Organization (Ibaraki, Japan). Each sample was cultured on a PDA (Merck, Darmstadt, Germany) slant, and incubated at 28 °C for several days. Spores were collected using 0.05% Tween 80 (ICN Biomedicals, Santa Ana, CA, USA), and stored at 4 °C.

### 5.2. Culture Conditions and Apparatus

To observe conidial formation, PDA plates without any additional reagents were first prepared. To observe AF-derived fluorescence, PDA supplemented with 3 g·L^−1^ α-CD (Wako Pure Chemical Industries, Osaka, Japan) and 0.3 g·L^−1^ activated charcoal powder (Neutral, Wako Pure Chemical Industries) was prepared. PD broth (Merck, Darmstadt, Germany) with activated carbon and without α-CD was also prepared as a liquid culture solution. Then, 495 μL of liquid medium was dispensed into 1-mL pipette tips, which were used as incubation apparatus (Figure S2), and the total amount was adjusted to 500 μL by adding the spore solution. Each spore solution (5 μL) was dispensed onto a culture plate or into the liquid culture medium. After liquid cultivation, the laboratory film (PM-996, Bemis, Neenah, WI, USA) wrapped around the top of each 1-mL tip was removed, and the tips were centrifuged at 2000× *g* for 10 min. The obtained culture solutions were used to measure the AF concentrations. The mycelia with spores in the incubation tips were dried in a dehydrator (SLI-450N, TOKYO RIKAKIKAI, Tokyo, Japan) at 65 °C overnight, and the fungal dry weight was measured by comparing the tip weight before and after incubation.

### 5.3. Light Conditions and Measurement of Fluorescence Intensity Levels

Light conditions were manually set up using shell-type light-emitting diodes on platforms (SPL-100-CC; Revox, Kanagawa, Japan). White, 401 nm, 470 nm, 503 nm, 525 nm, 548 nm, 570 nm, 595 nm, 660 nm, and 720 nm diodes (Figure S1) were selected for wavelengths in the visible region. The PFD was regulated by a pulse-width modulation dimmer controller. The spectrum of light-emitting diode radiation was measured by an illuminance spectrophotometer (CL-500A; Konica Minolta, Tokyo, Japan) as irradiance (W/m^2^). The total spectrum from 360 to 780 nm was counted, and the PFD of each spectral condition was calculated using the following formula: PFD (μmol·m^−2^·s^−1^) = [irradiance (W·m^−2^) × spectrum (m) × 10^−9^]/[Planck’s constant (6.626 × 10^−34^; J·s) × speed of light (2.998 × 10^8^; m·s^−1^) × Avogadro constant (6.022 × 10^23^; mol^−1^)] × 10^6^. Each culture sample, except under the dark condition, was continuously irradiated with 4 or 40 μmol·m^−2^·s^−1^ at different light wavelengths. The plates were incubated at 28 °C for two days, and the liquid culture media were incubated without shaking for three days. Two *Aspergillus* strains (*A. flavus* IFM55891 and *A. parasiticus* NRRL2999) were incubated on plain PDA plates, and four *Aspergillus* strains (*A. flavus* IFM55891, *A. parasiticus* NRRL2999, *A. bombysis* MAFFF111712 and *A. nomius* MAFF111739) were incubated on carbon-containing plates. As with plain PDA, the culture plates supplemented with α-CD and activated carbon were incubated for two days, and irradiated with UV light at 365 nm (UVGL-58, UVP, Upland, CA, USA) to confirm AF synthesis. The fluorescence signals were captured using a digital camera set to the manual white-balance setting (Caplio R3, Ricoh, Tokyo, Japan). The fluorescence intensity levels were extracted with digital image analysis software (ImageJ, version 1.40g, National Institutes of Health, Bethesda, MD, USA [27]). The changes in the fluorescence intensity levels were calculated using triplicate samples.

### 5.4. High-Performance Liquid Chromatography

Culture medium and an equivalent volume of chloroform were dispensed into new microtubes and vortexed for 10 s. The chloroform was collected and transferred to a new microtube, and then reduced to dryness in a draft chamber at 40 °C. Subsequent procedures followed the official method [28] (in Japanese. Officially, the limitations of quantitation and detection are 1 μg/kg and 0.5 μg/kg, respectively). For our instrument, the limitations of quantitation and detection were 2 μg/kg and 1 μg/kg, respectively. An aliquot of trifluoroacetic acid (Wako Pure Chemical Industries, Osaka, Japan) equal to 0.1 times the volume of chloroform was added to the microtube for derivatizing AFB_1_ and AFG_1_, and the tube was vortexed for 5 s. After incubation for more than 10 min, a solution of acetonitrile in distilled water (10:90, *v*/*v*) was added to the microtube, with the volume equal to 0.9 times the volume of chloroform. An aliquot (20 µL) of the sample solution was injected into a high-performance liquid chromatography system (SCL-10A, Shimadzu, Kyoto, Japan) with a fluorescence detector (λ_Ex_ = 365 nm and λ_Em_ = 455 nm; RF-535, Shimadzu, Kyoto, Japan). The mobile phase, which is a mixture of distilled water:methanol:acetonitrile (60:30:10, *v*/*v*/*v*), flowed through the column (Inertsil ODS-3, column size 4.6 × 150 mm, particle size 5 μm; GL Sciences, Tokyo, Japan) at a rate of 1 mL·min^−1^ to separate AFs.

### 5.5. Statistical Analyses

Average values of colony size, fluorescence intensity, fungal weight, and AF level were calculated, and the values under the dark condition were set as the control in each comparison. All comparison conditions were analyzed using an *F*-test, and then equally distributed conditions (*p* < 0.05) were analyzed using an unpaired *t*-test. For the unequally distributed conditions, Welch’s test was conducted. For the comparisons of AF concentrations, significant increases and decreases were discriminated, and indicated using plus or minus symbols, respectively. Each sample condition was prepared at least in triplicate.

## Figures and Tables

**Table 1:** Estimation of the light-radiation region that produces the most significant increase in AF synthesis.

	401	470	503	525	548	570	595	660	720	White
A	****	****	****	^1^ ++**	++**	****	****	****	****	****
B	^2^ − − −*	****	****	****	****	****	− ***	− ***	− − − *	****
C	^3^ ****	*+**	++*+	++**	*+*+	*+**	****	****	****	++**
D	++++	++++	++++	++++	++++	*+*+	++++	****	*+++	++++

^1^ A plus sign indicates a significant increase (*p* < 0.05) in aflatoxin synthesis. ^2^ A minus sign indicates a decrease (*p* < 0.05) in aflatoxin synthesis. ^3^ Asterisk; no significant change detected. The set of plus or minus signs in each cell reflects significant changes of AFB_1_, AFB_2_, AFG_1_, and AFG_2_, respectively. A; *A. flavus* IFM55891, B; *A. parasiticus* NRRL2999, C; *A. bombysis* MAFF111712, D; *A. nomius* MAFF111739.

## Supplementary Materials

The following are available online at www.mdpi.com/2072-6651/10/1/31/s1. Figure S1: Light spectra used in this study, Figure S2: Diagrammatic illustration of the modified tip-culture method, Figure S3: Changes in fungal dry mass after a three-day incubation period in liquid culture media at the 4 μmol·m^−2^·s^−1^ setting, Figure S4: Changes in fungal dry mass after a three-day incubation period in liquid culture media, Table S1: Aflatoxin concentrations per amount (mg·L^−1^) synthesized after a three-day incubation period in liquid culture media, Table S2: Aflatoxin concentrations per amount (mg·L^−1^) synthesized after a three-day incubation period in liquid culture media.
