# Supplementary material for: Light-Irradiation Wavelength and Intensity Changes Influence Aflatoxin Synthesis in Fungi
Source: Toxins (Basel). 2018 Jan 5;10(1):31. doi: 10.3390/toxins10010031 (PMC5793118; doi:10.3390/toxins10010031)
Supplement: Supplementary file 1 [file toxins-10-00031-s001.pdf]

Supplementary Materials: Light-Irradiation Wavelength and Intensity Changes Influence Aflatoxin Synthesis in Fungi

Tadahiro Suzuki

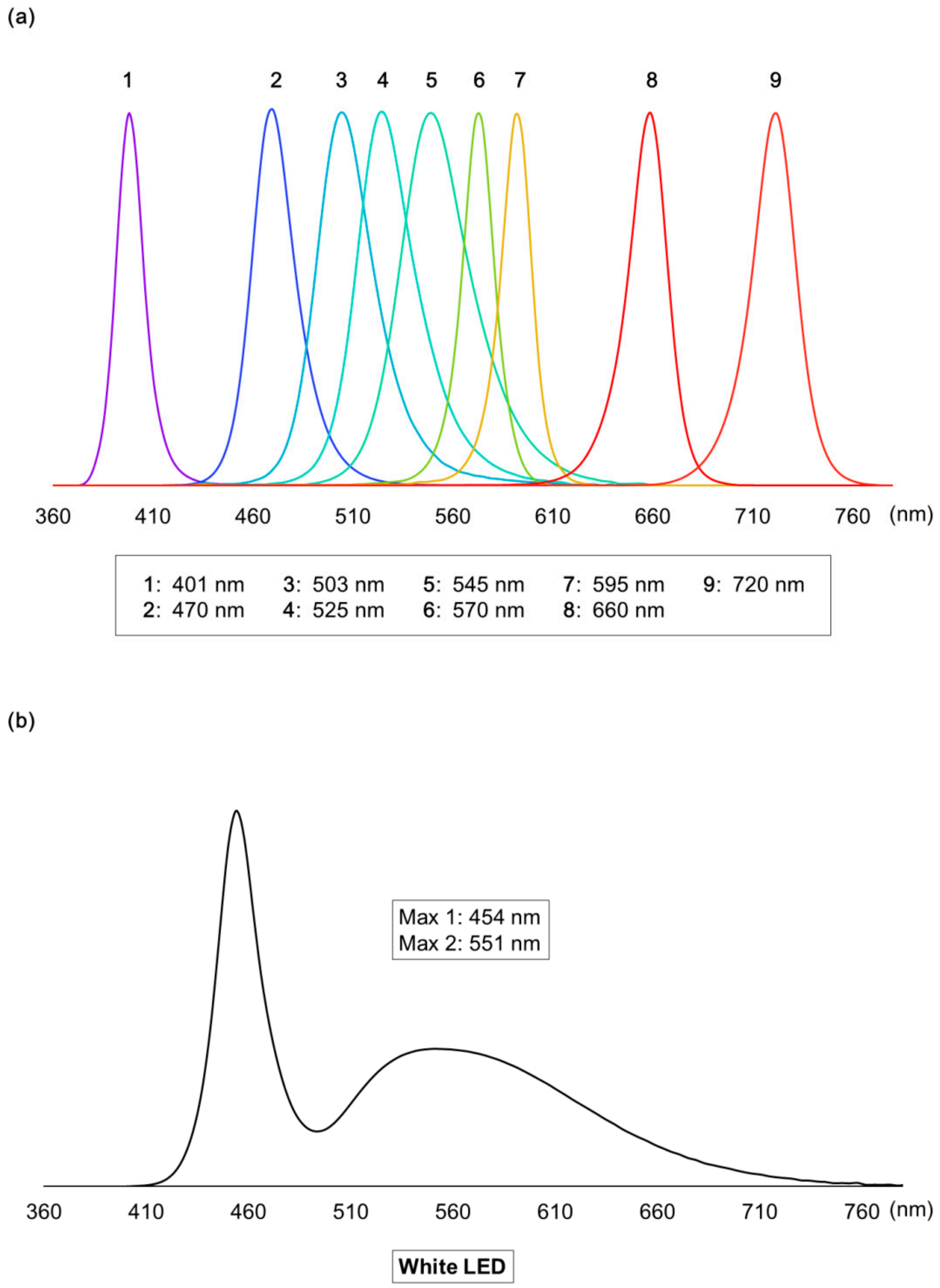

Figure S1. Light spectra used in this study. (**a**) Spectral characteristics of single wavelength light emitting diodes (LEDs). (**b**) Spectral characteristics of the white LED. Each spectrum was constructed by summing the spectral irradiances per 1 nm.

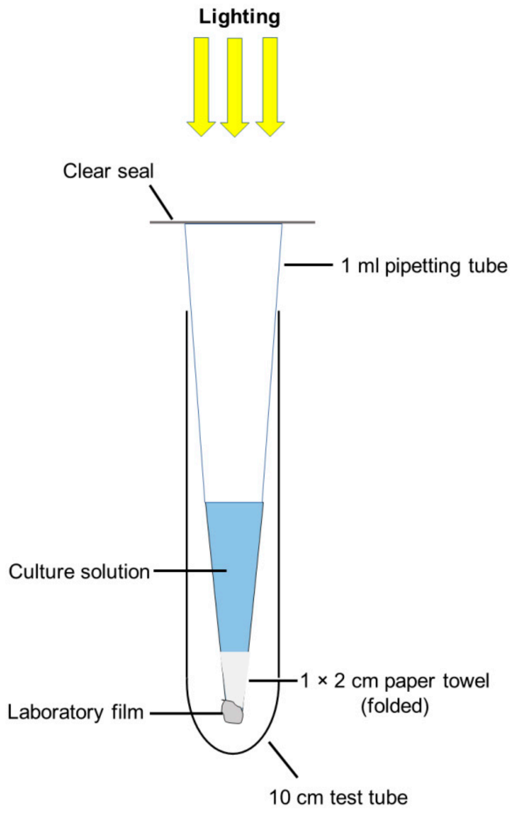

Figure S2. Diagrammatic illustration of the modified tip-culture method. The culture tip was placed under light irradiation at various settings and incubated without shaking. The light source was set at a distance of 15 cm from the surface of the culture solution.

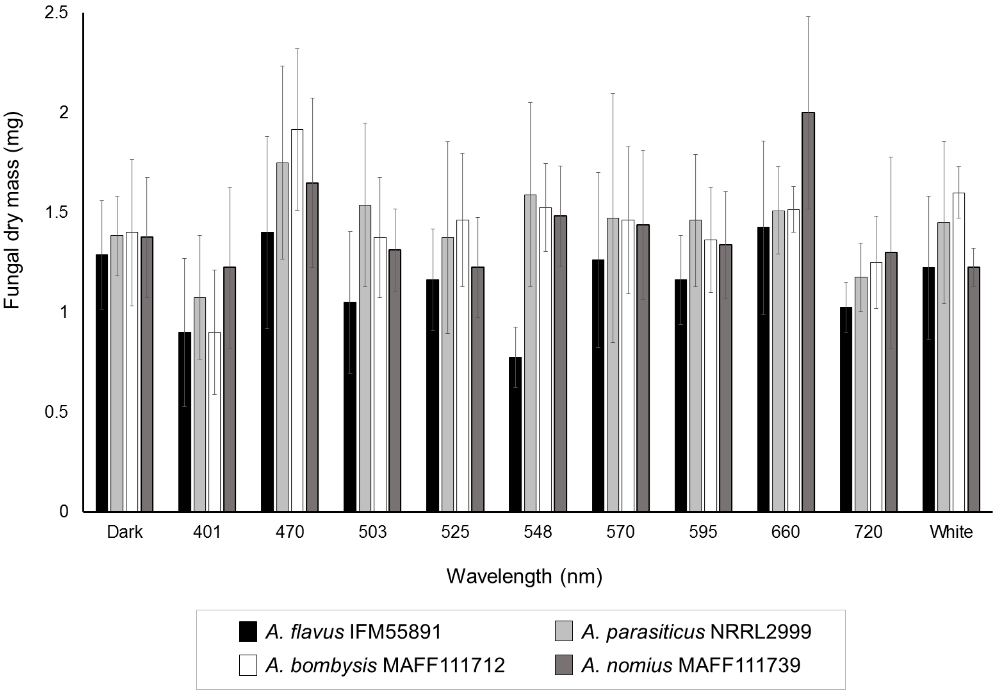

Figure S3. Changes in fungal dry mass after a 3-d incubation period in liquid culture media at the 4 μmol·m^{-2}·s^{-1} setting. Only 401, 548 and 720 nm for *A. flavus* IFM55891, 720 nm for *A. parasiticus* NRRL2999, and 660 nm for *A. nomius* MAFF111739 showed significant differences compared with dark conditions ($P < 0.05$). Bars indicate standard deviation; n = 3–6.

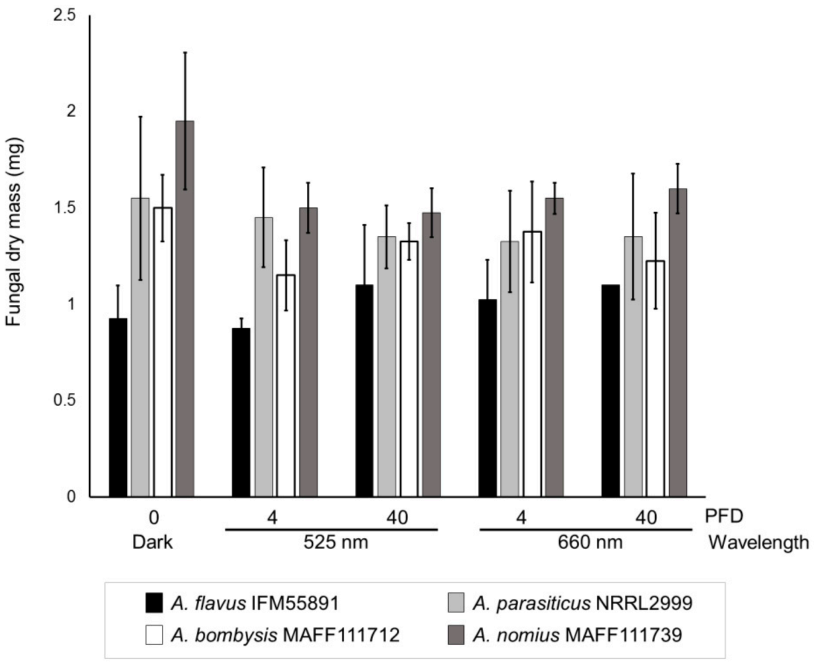

Figure S4. Changes in fungal dry mass after a 3-d incubation period in liquid culture media. Settings of 4 and 40 μmol·m^{-2}·s^{-1} at 525 and 660 nm were applied, except under dark conditions. Only 4 μmol·m^{-2}·s^{-1} at 525-nm conditions in *A. bombysis* MAFF111712 showed a significant decrease compared with dark conditions ($P < 0.05$). Bars indicate standard deviation; n = 4.

Table S1. Aflatoxin concentrations per amount (mg·L-1) synthesized after a 3-d incubation period in liquid culture media.

	**(nm)**	**AFG_1**	**AFB_1**	**AFG_2**	**AFB_2**
IFM55891	Dark	-	1.348 ± 0.292	-	0.037 ± 0.009
	401	-	0.994 ± 0.230	-	0.037 ± 0.007
	470	-	1.877 ± 0.239	-	0.057 ± 0.014
	503	-	1.974 ± 0.387	-	0.054 ± 0.019
	525	-	2.141 ± 0.277	-	0.060 ± 0.012
	548	-	1.900 ± 0.416	-	0.058 ± 0.013
	570	-	1.064 ± 0.157	-	0.032 ± 0.010
	595	-	1.383 ± 0.379	-	0.038 ± 0.009
	660	-	1.196 ± 0.229	-	0.038 ± 0.009
	720	-	1.946 ± 0.425	-	0.054 ± 0.003
	White	-	1.667 ± 0.426	-	0.056 ± 0.012
NRRL2999	Dark	6.521 ± 1.734	1.200 ± 0.450	0.163 ± 0.020	0.047 ± 0.009
	401	3.630 ± 0.967	0.388 ± 0.147	0.141 ± 0.020	0.023 ± 0.005
	470	6.721 ± 1.008	1.205 ± 0.394	0.193 ± 0.012	0.062 ± 0.014
	503	8.345 ± 1.909	1.616 ± 0.610	0.200 ± 0.055	0.063 ± 0.015
	525	7.131 ± 0.878	1.210 ± 0.367	0.203 ± 0.038	0.056 ± 0.008
	548	4.863 ± 0.976	0.691 ± 0.199	0.182 ± 0.033	0.043 ± 0.010
	570	3.861 ± 1.475	0.498 ± 0.258	0.152 ± 0.042	0.033 ± 0.011
	595	5.106 ± 1.218	0.676 ± 0.176	0.191 ± 0.036	0.040 ± 0.010
	660	4.670 ± 1.368	0.578 ± 0.158	0.191 ± 0.046	0.043 ± 0.010
	720	4.840 ± 1.954	0.490 ± 0.689	0.151 ± 0.022	0.026 ± 0.017
	White	7.302 ± 2.475	1.526 ± 0.812	0.189 ± 0.040	0.067 ± 0.025
MAFF111712	Dark	4.018 ± 0.231	1.943 ± 0.206	0.026 ± 0.002	0.028 ± 0.005
	401	2.375 ± 1.402	1.158 ± 0.937	0.035 ± 0.007	0.036 ± 0.021
	470	5.253 ± 2.300	3.710 ± 1.029	0.041 ± 0.016	0.055 ± 0.014
	503	5.301 ± 0.313	3.414 ± 0.459	0.042 ± 0.005	0.054 ± 0.006
	525	4.918 ± 0.484	3.224 ± 0.414	0.038 ± 0.004	0.055 ± 0.005
	548	5.315 ± 0.819	2.950 ± 0.576	0.043 ± 0.008	0.051 ± 0.009
	570	4.002 ± 0.567	1.906 ± 0.249	0.031 ± 0.002	0.033 ± 0.001
	595	3.302 ± 0.294	1.493 ± 0.051	0.026 ± 0.004	0.026 ± 0.002
	660	4.839 ± 0.378	2.372 ± 0.146	0.034 ± 0.006	0.033 ± 0.003
	720	4.990 ± 1.802	2.835 ± 0.785	0.026 ± 0.012	0.032 ± 0.010
	White	4.805 ± 0.390	3.213 ± 0.223	0.040 ± 0.002	0.050 ± 0.005
MAFF111739	Dark	0.347 ± 0.054	0.866 ± 0.074	0.001 ± 0.000	0.003 ± 0.001
	401	1.382 ± 0.547	1.382 ± 0.705	0.005 ± 0.002	0.017 ± 0.005
	470	2.207 ± 0.567	4.635 ± 1.072	0.007 ± 0.001	0.027 ± 0.004
	503	1.877 ± 0.283	4.804 ± 0.384	0.005 ± 0.001	0.027 ± 0.001
	525	1.790 ± 0.216	3.961 ± 0.202	0.005 ± 0.001	0.023 ± 0.005
	548	1.811 ± 0.204	3.787 ± 0.098	0.006 ± 0.001	0.021 ± 0.002
	570	1.299 ± 0.358	2.315 ± 0.836	0.006 ± 0.001	0.020 ± 0.007
	595	1.180 ± 0.348	1.727 ± 0.232	0.004 ± 0.001	0.008 ± 0.002
	660	0.831 ± 0.454	1.557 ± 0.748	0.002 ± 0.002	0.007 ± 0.004
	720	1.611 ± 0.192	2.235 ± 0.447	0.006 ± 0.000	0.012 ± 0.002
	White	1.783 ± 0.213	3.911 ± 0.630	0.007 ± 0.001	0.028 ± 0.001

Hyphen: no AF synthesis detected. Averages ± standard deviation. n = 3–6.

Table S2. Aflatoxin concentrations per amount (mg·L^{-1}) synthesized after a 3-d incubation period in liquid culture media.

		**PFD**	**AFG$_1$**	**AFB$_1$**	**AFG$_2$**	**AFB$_2$**
IFM55891	Dark	0	-	0.395 ± 0.086	-	0.010 ± 0.002
				525 nm		
		4	-	0.849 ± 0.171	-	0.024 ± 0.006
		40	-	0.699 ± 0.216	-	0.019 ± 0.009
				660 nm		
		4	-	0.534 ± 0.077	-	0.016 ± 0.003
		40	-	0.488 ± 0.170	-	0.012 ± 0.006
NRRL2999	Dark	0	6.806 ± 1.235	1.025 ± 0.273	0.219 ± 0.019	0.053 ± 0.006
				525 nm		
		4	6.848 ± 1.633	1.124 ± 0.516	0.182 ± 0.042	0.050 ± 0.007
		40	4.787 ± 1.235	0.910 ± 0.187	0.141 ± 0.034	0.049 ± 0.007
				660 nm		
		4	4.401 ± 0.373	0.732 ± 0.326	0.152 ± 0.049	0.041 ± 0.011
		40	4.634 ± 1.412	0.894 ± 0.432	0.163 ± 0.028	0.053 ± 0.013
MAFF111712	Dark	0	4.749 ± 0.697	2.615 ± 0.354	0.033 ± 0.004	0.035 ± 0.007
				525 nm		
		4	4.746 ± 0.488	3.725 ± 0.837	0.040 ± 0.005	0.059 ± 0.006
		40	4.527 ± 0.678	3.410 ± 0.584	0.044 ± 0.005	0.065 ± 0.005
				660 nm		
		4	4.693 ± 1.412	2.796 ± 0.823	0.038 ± 0.006	0.041 ± 0.010
		40	3.574 ± 0.669	2.286 ± 0.520	0.031 ± 0.006	0.034 ± 0.007
MAFF111739	Dark	0	0.990 ± 0.369	1.882 ± 0.555	0.003 ± 0.001	0.010 ± 0.004
				525 nm		
		4	1.803 ± 0.177	4.120 ± 0.174	0.008 ± 0.001	0.029 ± 0.002
		40	1.861 ± 0.365	4.545 ± 0.498	0.009 ± 0.002	0.030 ± 0.009
				660 nm		
		4	1.103 ± 0.366	2.638 ± 0.396	0.006 ± 0.001	0.016 ± 0.004
		40	0.919 ± 0.176	2.128 ± 0.369	0.005 ± 0.001	0.015 ± 0.003

Hyphen: no aflatoxin synthesis detected. Averages ± standard deviation. n = 4.
